# Standardized neutralization antibody analytical procedure for clinical samples based on the AQbD concept

**DOI:** 10.1038/s41392-023-01389-5

**Published:** 2023-04-28

**Authors:** Jianyang Liu, Yu Bai, Mingchen Liu, Dejiang Tan, Jing Li, Zhongfang Wang, Zhenglun Liang, Miao Xu, Junzhi Wang, Qunying Mao

**Affiliations:** 1 National Institutes for Food and Drug Control, Beijing, China; 2 NHC Key Laboratory of Research on Quality and Standardization of Biotech Products, Beijing, China; 3 NMPA Key Laboratory for Quality Research and Evaluation of Biological Products, Beijing, China; 4 Sinovac Life Science Co., Ltd., Beijing, China; 5 Guangzhou Laboratory, Guangzhou, Guangdong Province China

**Keywords:** Vaccines, Vaccines

**Dear Editor**,

Vaccination is an effective strategy for controlling the COVID-19 pandemic and reducing the number of cases of hospitalization and deaths.^1^ Neutralizing antibody (Ntab) is one of the key indicators for evaluating the effectiveness of COVID-19 vaccines.^2^ Over 40 COVID-19 vaccines have been approved for emergency use, marketing, or marketing with conditions, and the available Ntab data from clinical trials have been published.^3^ However, the lack of a standardized analytical procedure for Ntabs makes the Ntab detection results incomparable, limiting the development and application of COVID-19 vaccines. It has become an urgent issue for the World Health Organization and global vaccine regulatory agencies.

To solve this, a standardized Ntab analytical procedure for clinical samples was developed through the novel use of method development concepts, such as analytical quality by design (AQbD) and risk management according to USP < 1220 > and ICH Q14.^4,5^ Six key steps are described (Fig. 1a). The first step is to define the analytical target profile (ATP). Following discussions with six experienced professionals, this assay was developed for the Ntab detection of clinical samples, and the relative accuracy and intermediate precision were set within ±50% and within 100%, respectively (Supplementary Table 1). The method based on pseudotyped viruses expressing the Spike protein of SARS-CoV-2 has been developed to avoid using live virus and reduce the need for BSL-3 facilities. However, the live virus Ntab detection is still the gold standard. A survey of live virus Ntab assays was conducted in nine COVID-19 vaccine testing laboratories by official letter inquiries and literature review. It showed that the assays included the cytopathogenic efficiency (CPE) and plaque reduction neutralization test (PRNT). The Ntab test is mainly based on the CPE and is supplemented by PRNT (Supplementary Table 2). For high-throughput sample testing in the BSL-3 laboratory, CPE was chosen to establish the Ntab detection assay. According to the experimental processes, each step was analyzed to identify potential influencing factors that may affect the detection results.

**Fig. 1:**
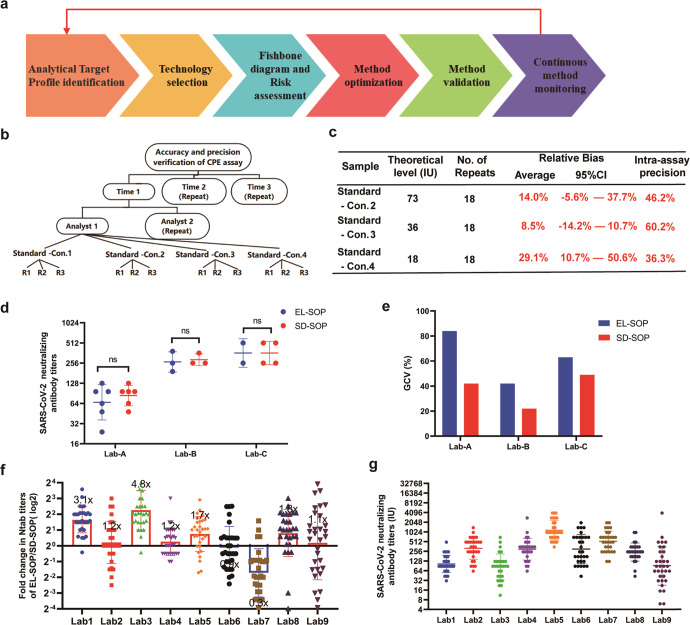
Development and application of standardized neutralization antibody analytical procedure. **a** Schematic diagram of the AQbD approach for analytical procedure development in this study. **b** Relative accuracy and intermediate precision validation were performed on 3 different days by two analysts. Each analyst prepared their own four samples and performed the procedures independently on each day. **c** Results are displayed in tables. **d** Ntab titers of the national standard tested in three laboratories using SD-SOP and EL-SOPs. **e** GCV of Ntab titers of national standard detected in three laboratories using SD-SOP and EL-SOPs. **f** Fold change of EL-SOP/SD-SOP in Ntabs titers. **g** Ntab levels of clinical samples from patients subjected to COVID-19 vaccines and convalescent serum marked by IU. **d**, **f**, **g** Bars represent geometric means, and error bars represent geometric standard deviations for each group. ANOVA-*t* test was used to compare the difference between both groups, and ns represents no significant difference

A total of 30 potential factors were identified and are listed in the fishbone diagram (Supplementary Fig. 1). Based on the ATP, the degree of influence of each factor on relative accuracy and intermediate precision was calculated. The influencing factors with a total score of 40 or more were considered the main risk factors; 13 main risk factors were obtained (supplementary Table 3). Among them, the two factors of “lesion judgment criteria” and “virus strain” were determined based on discussion, namely, the “lesion judgment criteria” took typical lesions caused by SARS-CoV-2 as the positive criteria, and the “virus strain” taking the selected original strain as the study object. Design of Experiments (DoE) is an important part of the assay development process. The remaining 11 factors were optimized using fixed-factor screening, single-factor experimental design, and custom-designed experiments. The experimental parameter settings for the optimization of the 11 main influencing factors are listed in supplementary Table 4. The type of cell-culture medium and time of post-neutralization incubation were first screened for fixed factors (Supplementary Fig. 2). Then, the conditions were optimized for the time after virus dilution and neutralization time using custom-designed experiments (Supplementary Fig. 3). Other factors, including the number of replicate wells, bovine serum concentration, number of cells per well, cell generation, virus addition method, and edge effect, were optimized using a single-factor experimental design (Supplementary Fig. 4). The optimization results of the 11 risk factors are summarized in Supplementary Table 5, including the suitable limit or range for the critical method parameters—time after virus dilution, number of cells per well, and cell generation.

After developing the Ntab assay, we validated the specificity, relative accuracy, and intermediate precision involving different analysts, time, and serial dilution of national standards to evaluate the method’s capability (Fig. 1b). Only the SARS-CoV-2 patient recovery serum had neutralizing activity against SARS-CoV-2 (Supplementary Table 6). The average relative bias of this assay ranged from 8.5 to 29.1%, and the geometric coefficient of variation (GCV) ranged from 36.3 to 60.2% (Fig. 1c), which met the ATP. The method capability was further evaluated using the validation data set. The prediction intervals and tolerance intervals corresponding to each concentration point of 73 IU/mL, 36 IU/mL, and 18 IU/mL were within the range of 25–400% (18.25–292, 9–144, 4.5–72 for 73 IU/mL, 36 IU/mL, and 18 IU/mL, respectively). The method capability indices all reached grade IV, and the maximus method variability was <65% (Supplementary Table 7). When the acceptance range of CQA for this assay was 25–400%, the method misjudgment rate was lower than 4.4%.

We further conducted an assay suitability study. Three clinical sample testing laboratories—A, B, and C—that have obtained China National Accreditation Service for Conformity Assessment certification have been approved by the National Health Commission of the People’s Republic of China to carry out BSL-3 experiments and have adopted their own SOP (each lab’s sop, EL-SOP). The Ntab titers of the national standard were 67, 266, and 362, and the GCV values were 84%, 42%, and 63%, respectively (Fig. 1d and e). When the developed assay (standardized SOP, SD-SOP) was adopted, the Ntab titers of the national standard were 84, 287, and 362, and the GCV values were 42%, 22%, and 49%, respectively (Fig. 1d and e). No significant differences in the Ntab titer of the national standard in each laboratory between EL-SOPs and SD-SOPs were observed. However, the intermediate precision of SD-SOP was significantly lower than that of EL-SOPs (Fig. 1e), indicating that SD-SOP is more robust than EL-SOP.

We collected 32 convalescent serum samples and 240 clinical samples from eight vaccines containing inactivated adenovirus vector, mRNA, and recombinant proteins in four technical routes. The SD-SOP established in this study was used to detect the 272 collected samples and national standards. When using EL-SOP, the Ntab titer detection results in lab1, lab3, lab5, and lab8 were 3.1-, 4.8-, 1.7- and 1.8- fold higher than that using SD-SOP, respectively. The Ntab titer detection results in Lab7 were 3.3-fold lower than that of SD-SOP (Fig. 1f). After converting to IU, the test results were 104, 319, 84, 297, 1162, 301, 524, 244, and 92 for labs 1–9, respectively. The Ntab levels were ordered as: lab5 > lab7 > lab2 > lab6 > lab4 > lab8 > lab1 > lab9 > lab3 (Fig. 1g). However, because the samples in this study are not representative, the results do not represent the Ntab levels of different vaccines in clinical trials. The above results showed that the Ntab assay established in this study, combined with the use of reference materials, could better unify the comparison standards and achieve a more objective, consistent, and robust evaluation of Ntab levels among different vaccines.

This study also has certain limitations. First, only the selected original virus strain was used in this study. Second, to ensure that the assay performs as robustly as the ATP throughout its life cycle, the reportable value of the national standard will be used for dynamic monitoring. Third, an international suitability study of this assay should be conducted in the future.

To sum up, this study introduced the concepts of AQbD, and risk identification, for the first time, for Ntab detection assay development. Combined with national standards, we provide a reliable and robust standardized detection platform for promoting COVID-19 vaccine development. Meanwhile, this study demonstrates the scientific and advanced nature of the AQbD concept and the innovation of the methodological capacity evaluation index, which can be applied to developing other assays.

## Supplementary information

Supplementary Materials for Standardized neutralization antibody analytical procedure for clinical samples based on the AQbD concept

Supplementary Fig. 1

Supplementary Fig. 2

Supplementary Fig. 3

Supplementary Fig. 4

## Data Availability

Data are available upon reasonable request.
