# Supplementary material for: Standardized neutralization antibody analytical procedure for clinical samples based on the AQbD concept
Source: Signal Transduct Target Ther. 2023 Apr 28;8:165. doi: 10.1038/s41392-023-01389-5 (PMC10140029; doi:10.1038/s41392-023-01389-5)
Supplement: Supplementary file 1 — Supplementary Materials for Standardized neutralization antibody analytical procedure for clinical samples based on the AQbD concept [file 41392_2023_1389_MOESM1_ESM.docx]

Supplementary Materials for

Standardized neutralization antibody analytical procedure for clinical samples based on the AQbD concept

Jianyang Liu, Yu Bai, Mingchen Liu, Dejiang Tan, Jing Li, Zhongfang Wang, Zhenglun Liang, Miao Xu, Junzhi Wang, Qunying Mao

Correspondence to: Xu Miao, xumiaobj@126.com; Wang Junzhi, wangjz@nifdc.org.cn; Mao Qunying, maoqunying@126.com

**This PDF file includes:**

Materials and Methods

**Supplementary Fig. 1**  to 4

**Supplementary** Tables 1 to 7

Materials and Methods

Materials

The COVID-19 virus sample was obtained from Sinovac Life Science Co., Ltd. (Beijing, China). Vero cells and Vero-E6 cells were CCL-81 and CRL-1586, respectively. Newborn calf serum (No.: SC111.02) was purchased from Lanzhou Minhai Biological Engineering Co., Ltd (Gansu, China). The 199 medium (No.: X103A6), DMEM medium (Art. No.: 11885-084), and MEM medium (No.: 11095-080) were purchased from Shanghai Basalmedia Technologies Co., Ltd (Shanghai, China) and Thermo Fisher Scientific (Waltham, MA, USA). Methods were established using high and low titers of convalescent plasma (XGR1 and XGR2) and COVID-19 vaccine goat immune serum (CGS). The specificity was validated using Influenza anti-A/Brisbane/02/2018-like (H1N1) HA Serum (Sheep 698, 699, 700, 701) 19/102, Influenza anti-A/Kansas/14/2017-like (H3N2) HA Serum 19/152, Influenza anti-B/Colorado/06/2017-like HA serum 18/170, Influenza Antiserum A/Vietnam/1194/04 (H5N1), and Non-WHO Reference Material Working reagent for anti-MERS-CoV antibody (NIBSC code: 19/180; MERS patient convalescent serum). SARS vaccine monkey immune serum, SARS vaccine human immune serum, and COVID-19 patient convalescent serum were provided by Sinovac Research & Development Co., Ltd.

The Chinese national standard for COVID-19 Ntabs (Lot: 280034-202102) used in this article was developed by the National Institutes for Food and Drug Control. The clinical serum of the vaccine and the convalescent serum of patients with COVID-19 detected in this study were provided by Sinovac Life Science Co., Ltd. (Beijing, China), Beijing Institute of Biological Products of China Pharmaceutical Group Co., Ltd. (Beijing, China), Wuhan Institute of Biological Products of China Pharmaceutical Group Co., Ltd. (Wuhan, China), Anhui Zhifei Longcom Biopharmaceutical Co., Ltd. (Hefei, China), Institute of Medical Biology of Chinese Academy of Medical Sciences (Yunnan, China), Shenzhen Kangtai Biological Products Co., Ltd. (Shenzhen, China), CanSino Biologics Inc. (Tianjin, China), and Walvax Biotechnology Co., Ltd (Kunming, China).

Determination of virus titer by the mixed-culture method

Cell maintenance solution (50 μL) was added to each well of the 96-well plate. The virus was serially diluted (10-fold) with sample or virus diluent to prepare eight concentrations ranging from 10^-3^ to 10^-10^. Virus solution (50 μL) was added to the 96-well plate, with eight wells for each concentration. Cell maintenance culture medium (100 μL) was added to the control wells. Each well was inoculated with 100 μL of cell suspension (10^5^ cells/mL) and cultured in an incubator at 37 ± 1 °C under 5% CO_2_ to observe cytopathic changes.

Determination of virus titer via the inoculation method

Cell suspension (100 μL; 10^5^ cells/mL) was added to each well of the 96-well plate and cultured in an incubator at 37 ± 1 °C under 5% CO_2_. After 48 h, the Vero cells grew into a thin monolayer, and the medium was discarded. The virus was serially diluted 10-folds using a cell maintenance culture medium. Diluted virus solution (50 μL) was added to the 96-well plate, with eight wells for each dilution. Cell maintenance solution (150 μL) was added to the sample wells. Cell maintenance solution (200 μL) was added to the control wells and cultured in an incubator at 37 ± 1 °C under 5% CO_2_ to observe cytopathic changes.

Neutralizing antibody detection methods

Cell maintenance culture medium (50 μL) was added to each well of the sample group. Serum samples were serially diluted (2-folds) in a 96-well plate with the sample and virus diluent. Each sample was diluted in duplicate. The internal, virus, and cell controls and virus back titration were established simultaneously. Virus suspension (50 μL) containing 100 CCID_50_ was added to the virus, internal control, and sample wells. Cell maintenance culture medium (100 μL) was added to the cell control wells and incubated at 37 ± 1 °C under 5% CO_2_. Cell suspension (100 μL) was added to each well and cultured at 37 ± 1 °C under 5% CO_2_ to observe the cytopathic changes and calculate neutralizing antibody titers and virus back titration.

Statistical analysis

For neutralizing antibody assays, the reciprocal of the highest dilution of samples with 50% cytopathic inhibition was used as the neutralizing antibody titer. The half-reaction volume (virus titer) of the neutralizing antibody results was calculated using Biostat®. The ANOVA-*t* test was used to calculate the significance of differences in neutralizing antibody levels between the two groups after log_2_ transformation. BMV® was used to calculate the intermediate precision, accuracy, and method ability evaluation index. GraphPad Prism 9.0® and JMP 13® were used to calculate and draft figures containing statistical analysis.

IU: when the neutralizing antibody of the serum sample was positive, the neutralizing titer was standardized to IU/mL, based on the titer of the national standard obtained from the same test, according to the following formula:

neutralizing antibody _sample to be tested_ (IU/mL) = neutralizing titer _sample to be tested_ / neutralizing titer _national standard_ × 580 IU/mL

**Supplementary Fig. 1**

Fishbone diagram of input factors with a potential impact on live virus Ntab assay of COVID-19 vaccines.

**Supplementary Fig. 2**

Fixed factor screening experiments. (**a**) Results of virus titer. (**b**) Geometric coefficient of variation (GCV) of virus titers. Bars represent geometric means, and error bars represent geometric standard deviations for each group.

**Supplementary Fig. 3**

Customized experiments.

(**a**) Customized experimental design for use time after virus dilution and neutralization time. (**b**) Ntab levels of the CGS sample and virus back titration results. (**c**) Ntab levels of XGR1 samples and virus back titration results. Bars represent means, and error bars represent 95% confidence interval for each group.

**Supplementary Fig. 4**

Optimization of single factor experiments.

(**a, b, and d**) Results of virus titer. (**c and e**) GCV of virus titers. (**f and g)** Ntab levels of XGR1 and XGR2 at different number of cells per well. (**h**) Ntab levels of CGS, XGR1, and national standards with different numbers of replicate wells. (**i**) GCV for Ntab levels of CGS, XGR1, and national standards in different replicate wells. (**j**) Schematic diagram of the middle and edge well positions. (**k**) Statistical results of the proportion of cytopathic wells at different dilutions of XGR1 and CGS. (**a, b, d, and h**) Bars represent means, and error bars represent 95% confidence interval for each group. ANOVA-*t* test was used to compare the differences. ns represents non-significant differences. (**k**) The chi-square test was used to compare the differences between the number of cytopathic and non-cytopathic pores. (p-value: *<0.05, ***<0.001)

**Supplementary Table 1.** ATP of COVID-19 Ntab assay

|  | | **Principle** | **Acceptance criteria** | **Validation results** |
| --- | --- | --- | --- | --- |
| **Intended use** | | This assay was used to determine the potency of Ntab against SARS-CoV-2 in clinical samples of COVID-19 vaccine. | | |
| **CQA for testing** | | SARS-CoV-2 Ntab specifically binds to SARS-CoV-2, preventing it from infecting cells and inhibiting the appearance of cytopathic lesions. | | |
| **Performance attributes** | **Specificity** | Specific neutralization of SARS-CoV-2 in samples | Negative neutralization reaction against pathogens except against SARS-CoV-2 | Negative neutralization reaction against pathogens except against SARS-CoV-2 |
|  | **Relative accuracy** | Accuracy examines the closeness of the test results of the Ntab level to the true value of the international standard. Average relative bias was used to assess the accuracy | Average relative bias: absolute value <50% | Average relative bias: absolute value <30% |
|  | **Intermediate precision** | The precision between the results of multiple people testing the same sample on multiple different days using this assay was assessed using geometric coefficient of variation (GCV) | GCV <100% | GCV <65% |

CQA: critical quality attribute

**Supplementary Table 2.** Summary of main clinical Ntab live virus detection assays for COVID-19 vaccines

| **Lab** | **Vaccine** | **Method** | **Detected virus strain** | | **Cell** | | | **Medium** | **Bovine serum concentration** | **Neutralization conditions** | **Incubation time (day)** |
| --- | --- | --- | --- | --- | --- | --- | --- | --- | --- | --- | --- |
|  |  |  | **Name (WT)** | **Challenge dose (CCID_50_/ well)** | **Name** | **Quantity (unit/well)** | **Generation** |  |  |  |  |
| 1 | Vaccine-A | CPE | / | 100 | Vero | (1–2)×10^4^ | / | 199 | 6% NBCS | 36.5 ℃, 2 h | 3–5 |
| 2 | Vaccine-B | CPE | / | 100 | Vero | (1–1.5)×10^4^ | Passing on 3–6 days | DMEM | 2.5-5% | 37.0 ℃, 2 h | 5–7 |
| 3 | Vaccine-C | PRNT | / | 180 PFU | Vero | (3.0–5.0)×10^5^ | <150 | DMEM | 2.5% NBCS | 37.0 ℃, 1 h | 3–5 |
| 4 | Vaccine-D | CPE | SARS-CoV-2/19nCoV-CDC-Tan-Strain04, QD01 | 100 | Vero | (1.5–2.5)×10^4^ | / | / | / | 37.0 ℃, 2 h | 4 |
| 5 | Vaccine-E | CPE | SARS-CoV-2 | / | / | / | / | / | / | / | / |
| 6 | Vaccine-F | CPE | SARS-CoV-2/CAS-B001/2020 (P3-5) | 100 | Vero- E6 | (1.2–1.8)×10^4^ | Passing on 10–15 days | DMEM | 7.5% FBS | 37 ℃, 2 h | 3 |
| 7 | Vaccine-G | CPE | SARS-CoV-2/  human/CHN/Wuhan_IME-BJ01/2020 | / | / | / | / | / | / | / | / |
| 8 | Vaccine-H | CPE | SARS-CoV-2 virus strain BetaCoV/Jiangsu/JS02/2020 (EPI_ISL_411952) | 200 TCID_50_ | Vero- E6 | / | / | DMEM | 6% FBS | 37 ℃, 1 h | 3–5 |
| 9 | Vaccine-I | PRNT | Patient-derived SARS-CoV-2 isolates (Beijing) | 200 PFU/mL  0.25 mL | Vero | 2×10^5^ | / | DMEM | 2% FBS | 37 ℃, 1 h | 2 |

PFU: plaque-forming units. TCID50: tissue culture infective dose 50. FBS: fetal bovine serum. NBCS: newborn calf serum. DMEM: Dulbecco's Modified Eagle Medium.

**Supplementary Table 3.** Risk analysis of factors influencing the SARS-CoV-2 Ntab assay

| **ID** | **Step** | **Step explanation** | **Relative accuracy (10)** | **Intermediate precision (10)** | **Total score** |
| --- | --- | --- | --- | --- | --- |
| 1 | Number of replicate wells for each dilution | Number of repeat holes: 2, 4, 8 | 5 | 5 | 100 |
| 5 | Virus strains | Different laboratory sources | 4 | 4 | 80 |
| 8 | Time after virus dilution | Use time after virus dilution | 3 | 3 | 60 |
| 12 | Types of cell-culture media | Vero, Vero-E6 (Contains adapted media types） | 5 | 1 | 60 |
| 20 | Bovine serum concentration of cell maintenance culture | Cell maintenance fluid: different concentrations of bovine serum | 5 | 1 | 60 |
| 27 | Time of post-neutralization incubation | Number of days of incubation after neutralization | 4 | 2 | 60 |
| 28 | Lesion judgment criteria | Lesion wells: >50% and >0% | 4 | 2 | 60 |
| 29 | Edge effect | 96-well plate sample sorting | 3 | 3 | 60 |
| 4 | Bovine serum concentration of samples, virus diluent | Sample and virus dilutions: different concentrations of bovine serum | 3 | 2 | 50 |
| 11 | Neutralization time | Different lengths of neutralization reaction time | 4 | 1 | 50 |
| 13 | Cell dosage | Different number of inoculated cells per well | 3 | 1 | 40 |
| 14 | Cell generation | High and low cell generations | 3 | 1 | 40 |
| 26 | Virus addition method | Mixing or inoculation | 3 | 1 | 40 |
| 2 | Sample extinction | Whether to extinguish energy | 2 | 2 | 30 |
| **......** |  |  |  |  |  |

**Supplementary Table 4.** Parameter settings of optimization experiments

| **Risk factor** | Unit | **Level** | | | | | |
| --- | --- | --- | --- | --- | --- | --- | --- |
|  |  | Level 1 | Level 2 | Level 3 | Level 4 | Level 5 | Level 6 |
| Number of replicate wells for each dilution | Well | 2 | 4 | 8 | - | - | - |
| Bovine serum concentration of sample and virus diluent | % | 2 | 10 | - | - | - | - |
| Time after virus dilution | H | 0 | 1 | 2 | - | - | - |
| Neutralization time | H | 1 | 2 | 3 | - | - | - |
| Culture-cell type | N/A | 199-Vero | DMEM-Vero | MEM-Vero | 199-Vero-E6 | DMEM-Vero-E6 | MEM-Vero-E6 |
| No. of cells per well | 10^4^ cells/well | 1.0 | 2.0 | 3.0 | - | - | - |
| Cell generation | Generation | 143 | 150 | 155 | 160 | - | - |
| Bovine serum concentration of cell maintenance culture | % | 2 | 10 | - | - | - | - |
| Virus addition method | N/A | mix | inoculation | - | - | - | - |
| Time of post-neutralization incubation | D | 2 | 3 | 4 | 5 | - | - |
| Edge effect | N/A | edge | middle | - | - | - | - |

**Supplementary Table 5.** Summary results of the Ntab assay optimization

| **ID** | **Step** | **Procedure** | **Experimental conclusion** |
| --- | --- | --- | --- |
| 1 | Pre-experimental design | Repeat wells | 2 wells |
| 4 | Step 1: Sample preparation | Bovine serum concentration of samples and virus diluent | 2% |
| 8 | Step 2: Virus preparation and virus addition | Time after virus dilution | Use immediately after dilution, maximum 1 h |
| 11 | Step 3: Antibody and virus neutralization | Neutralization time | 2 h |
| 12 | Step 4: Cell preparation and addition | Cell type, culture medium type | Vero cell, 199 medium |
| 13 | Step 4: Cell preparation and addition | No. of cells per well | (1.0–2.0)×10^4^ cells/well |
| 14 | Step 4: Cell preparation and addition | Cell generation | 143–155 |
| 20 | Step 4: Cell preparation and addition | Bovine serum concentration of cell maintenance culture | 10% |
| 26 | Step 4: Cell preparation and addtion | Virus addition method | mixed |
| 27 | Step 5: Cell culture and result counts after neutralization | Time of post-neutralization incubation | Day 5 |
| 29 | Step 5: Post-neutralization cell culture and result readout | 96-well plate sample sorting | No edge effect was found |

**Supplementary Table 6.** Specificity validation

| **Sample Name** | **Sample lots** | **Results**  **（1:X）** | **Acceptable standards** | **Conformity with acceptable standards or not** |
| --- | --- | --- | --- | --- |
| Recovery serum for patients infected with SARS-CoV-2 | / | 96 | Positive | Yes |
| SARS vaccine monkey immunization serum | / | <4 | Negative | Yes |
| SARS vaccine human immune serum | / | <4 | Negative | Yes |
| MERS patient convalescent serum | MERS S-1 | <4 | Negative | Yes |
| MERS patient convalescent serum | MERS S-2 | <4 | Negative | Yes |
| Influenza anti-A/Brisbane/02/2018-like (H1N1) HA Serum (Sheep 698, 699, 700, 701) 19/102 | / | <4 | Negative | Yes |
| Influenza anti-A/Kansas/14/2017-like (H3N2) HA Serum 19/152 | / | <4 | Negative | Yes |
| Influenza anti-B/Colorado/06/2017-like HA serum 18/170 | / | <4 | Negative | Yes |
| Influenza Antiserum A/Vietnam/1194/04 (H5N1) | / | <4 | Negative | Yes |

MERS: middle east respiratory syndrome.

**Supplementary Table 7.** Method capability evaluation

| **Concentration**  **(IU/mL)** | **Method**  **Variability (%)** | **90% tolerance**  **interval** | **90% prediction**  **interval** | **MCI**  **under** | **Method misjudgment**  **rate lower than** | **Method level** |
| --- | --- | --- | --- | --- | --- | --- |
| 73 | 48.3% | 32.2–215.3 | 40.6–170.6 | 0.797 | 1.7% | IV |
| 36 | 60.8% | 12.6–121.0 | 16.6–91.7 | 0.670 | 4.4% | IV |
| 18 | 46.5% | 9.0–60.1 | 11.3–47.7 | 0.798 | 1.7% | IV |

MCI: method capability index
