# Supplementary figures and images for: Standardized neutralization antibody analytical procedure for clinical samples based on the AQbD concept
Source: Signal Transduct Target Ther. 2023 Apr 28;8:165. doi: 10.1038/s41392-023-01389-5 (PMC10140029; doi:10.1038/s41392-023-01389-5)

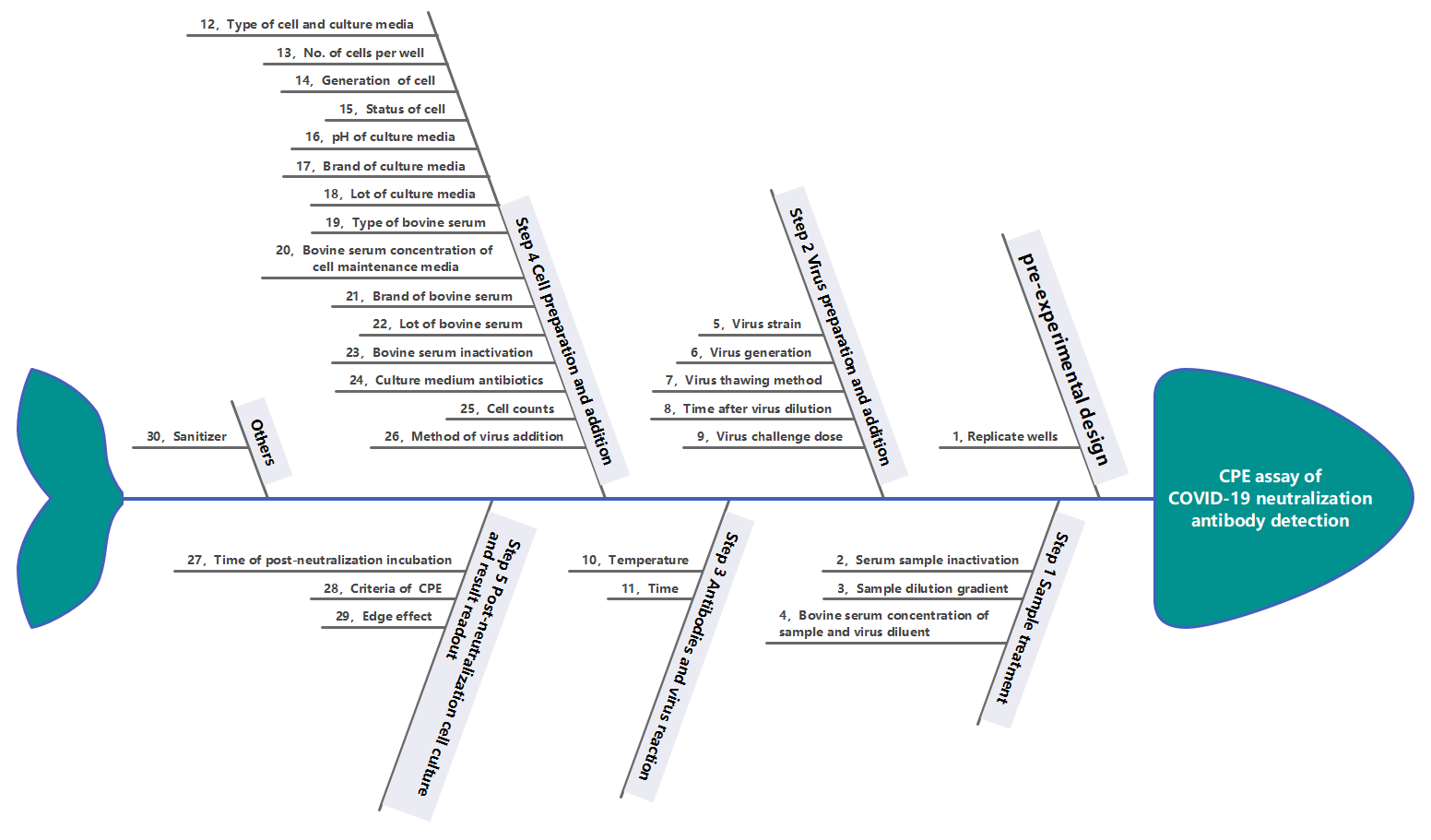

Supplement: Supplementary file 2 — Supplementary Fig. 1 [file 41392_2023_1389_MOESM2_ESM.tif]

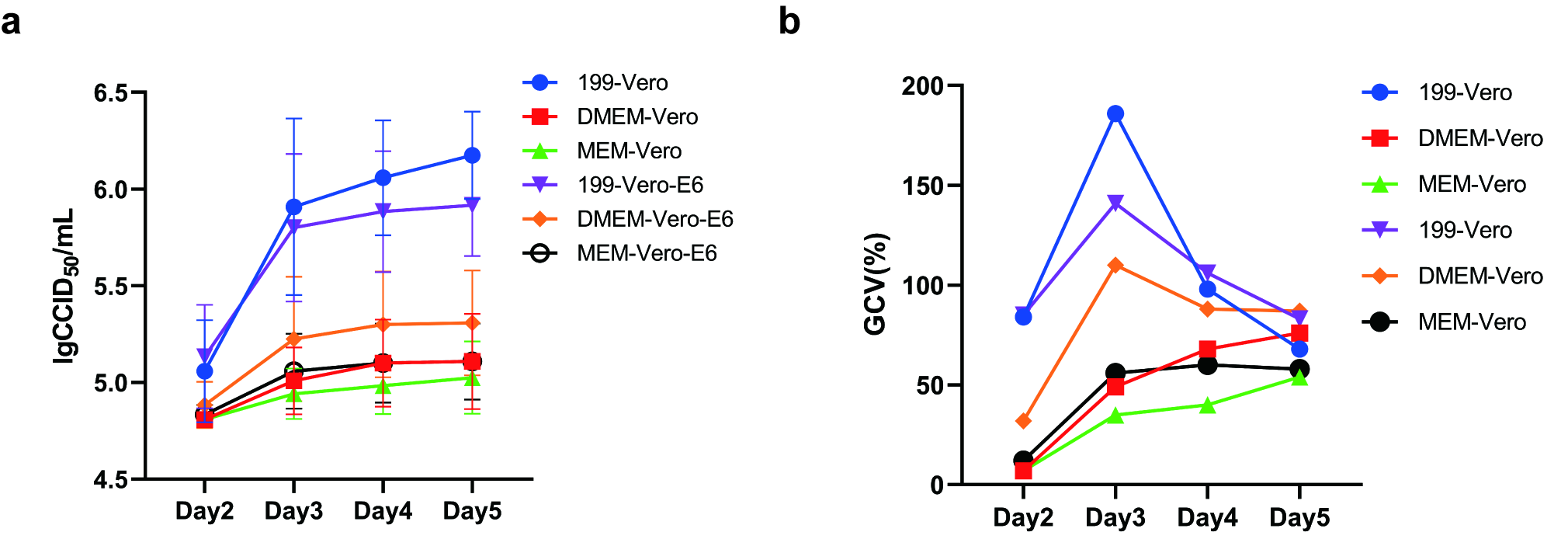

Supplement: Supplementary file 3 — Supplementary Fig. 2 [file 41392_2023_1389_MOESM3_ESM.tif]

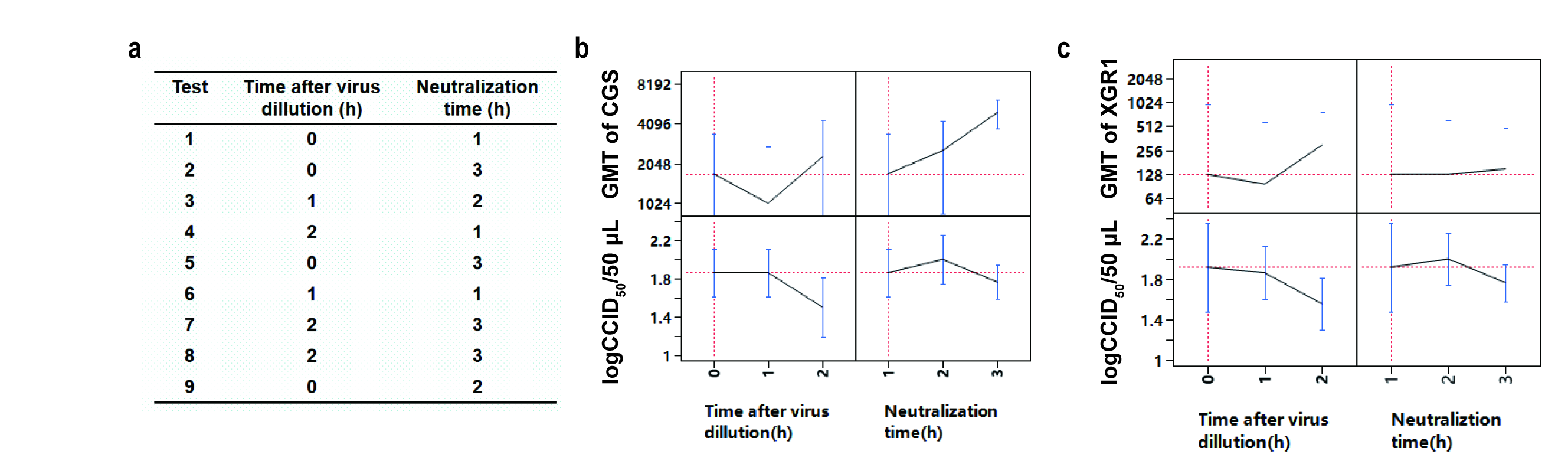

Supplement: Supplementary file 4 — Supplementary Fig. 3 [file 41392_2023_1389_MOESM4_ESM.tif]

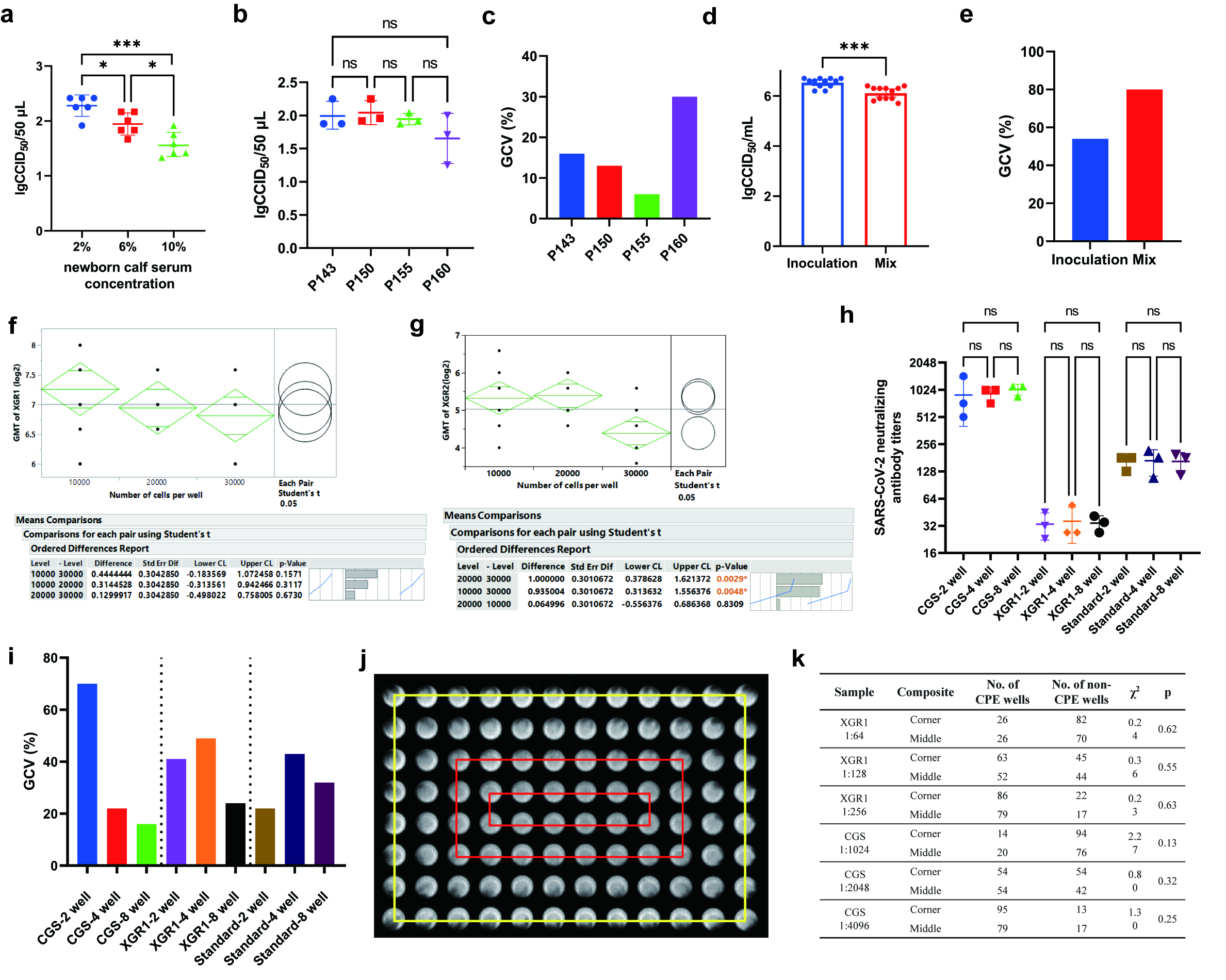

Supplement: Supplementary file 5 — Supplementary Fig. 4 [file 41392_2023_1389_MOESM5_ESM.tif]
